# Laparoscopic transabdominal preperitoneal repair of a hydrocele of the canal of Nuck in an adult woman

**DOI:** 10.1093/jscr/rjaf1081

**Published:** 2026-01-16

**Authors:** Andrew Stafford Beatty, Isidora De La Cruz, Kasra Raufian

**Affiliations:** Department of General Surgery, Redcliffe Hospital, Redcliffe 4020, Queensland, Australia; Northside Clinical School, School of Medicine, The University of Queensland, Brisbane, QLD, Australia; Department of General Surgery, Redcliffe Hospital, Redcliffe 4020, Queensland, Australia; Northside Clinical School, School of Medicine, The University of Queensland, Brisbane, QLD, Australia; Department of General Surgery, Redcliffe Hospital, Redcliffe 4020, Queensland, Australia

**Keywords:** canal of Nuck, hydrocele, laparoscopic, trans-abdominal prePeritoneal, TAPP

## Abstract

Hydrocele of the canal of Nuck is a rare pathology seen in adult women. Homologous with the processus vaginalis in men, pathologies of the canal of Nuck arise from a failure of obliteration of this tract. More commonly they present in children but rarely manifest in adults typically presenting as a painful lump which can be difficult to distinguish clinically from hernias. Cross-sectional imaging is key in diagnosis and surgery is the mainstay of treatment as it allows of excision of the hydrocoele and reinforcement of the defect to prevent subsequent hernias. While often the pathology is benign, malignancies have been documented and once excised the hydrocele should undergo pathological analysis to exclude such malignancies. Herein we present the case of a 44-year-old women who presented with a symptomatic hydrocele of the canal of Nuck confirmed on imaging that was successfully treated with a laparoscopic transabdominal preperitoneal approach.

## Introduction

Swellings in the groin are a common complaint prompting a general surgical referral, yet the causes are vast and pathologies of the canal of Nuck represent one of the rarer differentials. These pathologies are mostly seen in younger girls and rarely present in adult women [1]. Most cases it reflects a benign process, but malignancies have been reported [2] hence surgical excision is the mainstay of treatment allowing for histological analysis. Herein we present the case of an adult woman who presenting with a palpable groin lump that was diagnosed as a hydrocele of the canal of Nuck and was successfully treated with laparoscopic transabdominal preperitoneal (TAPP) approach.

## Case report

A 44-year-old woman was referred to the surgical outpatients after noticing a right groin lump that was increasing in size over a 4-month period. There was associated discomfort that was exacerbated with exercise and prolonged standing however upon laying supine the lump fully reduced, and the pain subsided. The lump became readily apparent upon standing whereby the patient describes a gushing or filling sensation and within a minute the lump would become visible.

She had no significant past medial history and had no previous operations. She had 4 children all born vial spontaneous vaginal deliveries. She took no regular medications.

On examination she was of normal habitus (BMI 30) and there was a mildly tender fluctuant lump overlying the right superficial inguinal ring that was apparent upon standing but spontaneously reduced upon laying supine. There was no cough impulse and no contralateral masses nor inguinal lymphadenopathy.

**Figure 1:**
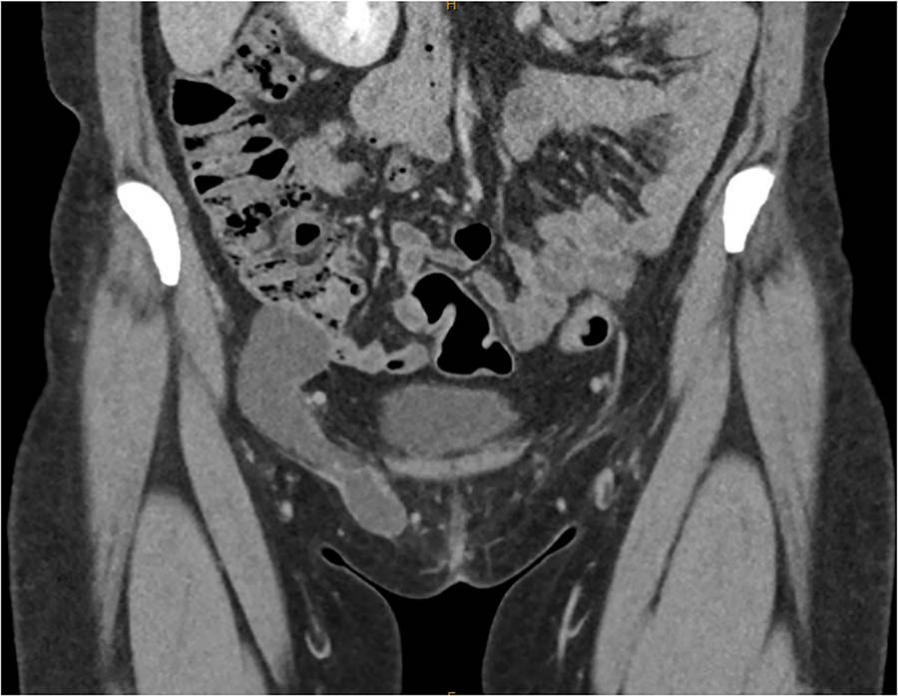
Coronal slice of CT demonstrating the bilobar right sided hydrocele of the canal of Nuck.

She had a computed tomography (CT) abdomen and pelvis with straining phase which demonstrated a bilobar cystic structed extending through the inguinal ring. The peritoneal component measured 46 × 35 × 33 mm and the inguinal measured 76 × 13 × 20 mm. There was no herniation of any intraperitoneal contents thus confirming the diagnosis of a hydrocele of the canal of Nuck (Fig. 1).

**Figure 2:**
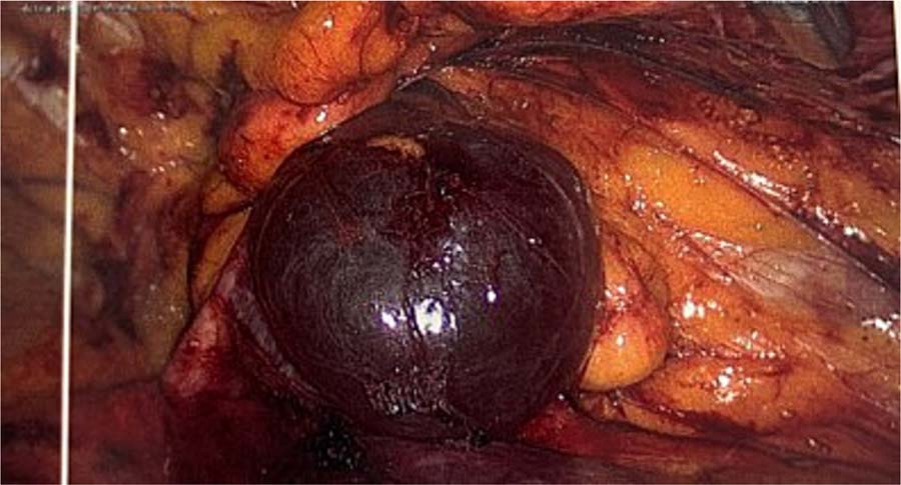
Intraoperative image of the hydrocele (instrument overlying the inferior epigastric vessels.

**Figure 3:**
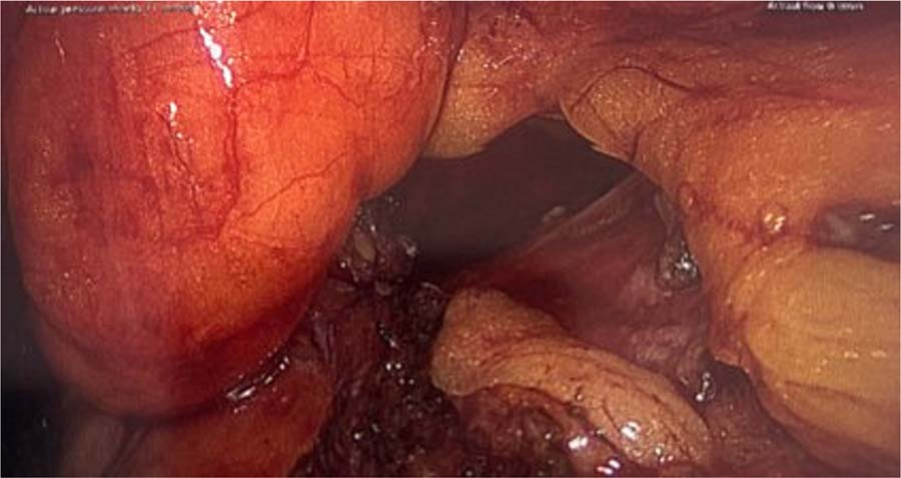
Intraoperative picture following excision of the hydrocele demonstrating the resulting defect.

**Figure 4:**
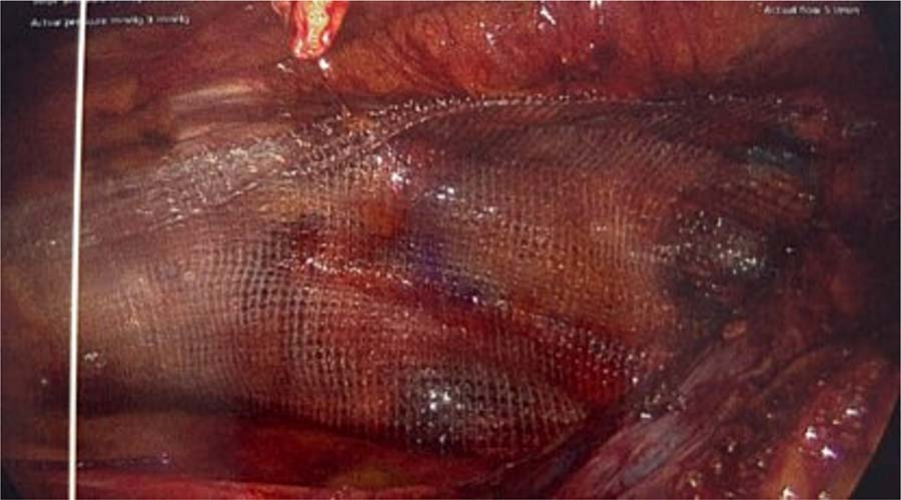
Intraoperative picture showing the progrip mesh *in situ* now covering the defect.

She proceeded for a laparoscopic TAPP excision of the hydrocele. After the abdominal cavity was entered and insufflated the preperitoneal flap was raised the hydrocele was identified medial to the inferior epigastric vessels (Fig. 2). With medial traction the hydrocele was able to be fully reduced and dissected free from the round ligament. The hydrocele was retrieved and sent for histological review. The resultant defect following excision (Fig. 3) was re-enforced with a 12 × 16 cm ProGrip mesh (Fig. 4) before the closure. She was observed post operatively and discharged home on the same day as her surgery.

The histology of the resected hydrocele demonstrated a single layer of mesothelium with interspaced mature adipose tissue. There was no evidence of dysplasia or malignancy.

She was reviewed in the outpatient clinic 6 weeks later with complete resolution of her symptoms and other than some initial post operative pain had no complication for surgery and was successfully discharged.

## Discussion

Hydrocele of the canal of Nuck was first described in 1691 by Anton Nuck and represents a rare differential for a women presenting with a groin lump [3]. The canal of Nuck is homologus with the processes vaginalis in males and normally, the canal should be obliterated before the first year of life [1]. However, failure of obliteration results in various pathologies which can range from an encysted hydrocele to herniation of abdominal contents such as the uterus or ovary [4].

Hernias associated with the canal of Nuck occur due to the patent processus vaginalis however these hernias carry a high morbidity due to the risk of incarceration which can occur in both pediatric and adult cases [5, 6].

Radiological assessment is crucial in the assessment as it allows for the differentiation of other pathologies such as inguinal hernias, leiomyomas, or lymph node metastases [7]. Several imaging modalities have been described including ultrasound, CT, and magnetic resonance imaging (MRI). Ultrasound while usually readily available has limitations in its evaluation of deeper structures thus cross section imaging is recommended. Often CT is preferred as it is less expensive and often more available than MRI [8].

Surgical excision remains the mainstay of treatment with both open and laparoscopic approaches having been described. Due to the rarity of the condition there are no comparative studies on laparoscopic versus open approaches though authors have drawn parallels from the outcomes of laparoscopic versus open repair for inguinal hernias referencing less post operative pain, shorter inpatient length of stay and quicker recovery from surgery [7]. Regardless, an open anterior approach is recognized and can be considered in patients where laparoscopy is contraindicated, and it offers easy access to the distal component of the cyst/sac assisting with a complete sac excision.

The choice of Totally Extra-Peritoneal versus TAPP repair is determined by the surgeon’s preference although some authors comment that TAPP offers more diagnostic information which can be helpful if there is concern for a concurrent hernia [9]. Some have proposed that TAPP offers a lower risk of conversion to open with better exposure of the inferior epigastric vessels and, due to the more lateral port placements, helps with the separation of the cyst from the vessels reducing the bleeding risk [1, 7, 9].

In up to one third of cases there is an associated inguinal hernia due to the widening of the internal ring [3]. Additionally, to excise the cyst intact may require widening of the neck hence Venkateswaran *et al*. [7] recommended that the posterior wall should be reinforced with a mesh regardless of the present of a hernia or not due to the future risk of herniation. In this case following excision of the sac this defect is clearly visible (Fig. 3) and would be expected to develop into a hernia if left untreated.

## Conclusion

Hydrocele of the canal of Nuck is a rare but important differential for a woman with a groin lump. Imaging plays a key role in the diagnosis, but definitive management requires excision of the sac which should be sent for histology. Following excision there should be consideration to re-enforce the resultant defect thereby reducing the risk of hernia development later.
